# Supplementary material for: Modeling glioblastoma heterogeneity as a dynamic network of cell states
Source: Mol Syst Biol. 2021 Sep 16;17(9):e10105. doi: 10.15252/msb.202010105 (PMC8444284; doi:10.15252/msb.202010105)
Supplement: Supplementary file 5 — Source Data for Figure 3 [file MSB-17-e10105-s001.zip › Figure3A_sourcedata/GSEA_3065/hallmarks_state6.GseaPreranked.1623416308702/gsea_report_for_na_pos_1623416308702.html]

Report for na\_pos 1623416308702 [GSEA]

| GS  follow link to MSigDB | GS DETAILS | SIZE | ES | NES | NOM p-val | FDR q-val | FWER p-val | RANK AT MAX | LEADING EDGE || 1 | HALLMARK\_P53\_PATHWAY | Details ... | 132 | 0.57 | 2.53 | 0.000 | 0.000 | 0.000 | 1005 | tags=34%, list=11%, signal=38% |
| 2 | HALLMARK\_OXIDATIVE\_PHOSPHORYLATION | Details ... | 186 | 0.35 | 1.61 | 0.000 | 0.038 | 0.088 | 1578 | tags=30%, list=18%, signal=35% |
| 3 | HALLMARK\_TNFA\_SIGNALING\_VIA\_NFKB | Details ... | 103 | 0.36 | 1.51 | 0.017 | 0.068 | 0.219 | 1529 | tags=31%, list=17%, signal=37% |
| 4 | HALLMARK\_MYC\_TARGETS\_V1 | Details ... | 194 | 0.29 | 1.35 | 0.012 | 0.183 | 0.608 | 1200 | tags=20%, list=14%, signal=23% |
| 5 | HALLMARK\_TGF\_BETA\_SIGNALING | Details ... | 45 | 0.37 | 1.34 | 0.069 | 0.166 | 0.655 | 1921 | tags=33%, list=22%, signal=42% |
| 6 | HALLMARK\_APOPTOSIS | Details ... | 106 | 0.30 | 1.27 | 0.071 | 0.232 | 0.829 | 744 | tags=19%, list=8%, signal=20% |
| 7 | HALLMARK\_WNT\_BETA\_CATENIN\_SIGNALING | Details ... | 27 | 0.39 | 1.26 | 0.151 | 0.223 | 0.857 | 595 | tags=15%, list=7%, signal=16% |
| 8 | HALLMARK\_HEME\_METABOLISM | Details ... | 116 | 0.27 | 1.17 | 0.133 | 0.344 | 0.975 | 1436 | tags=26%, list=16%, signal=30% |
| 9 | HALLMARK\_ALLOGRAFT\_REJECTION | Details ... | 60 | 0.30 | 1.17 | 0.208 | 0.319 | 0.977 | 759 | tags=20%, list=9%, signal=22% |
| 10 | HALLMARK\_DNA\_REPAIR | Details ... | 123 | 0.26 | 1.15 | 0.179 | 0.324 | 0.987 | 2200 | tags=34%, list=25%, signal=45% |
| 11 | HALLMARK\_UV\_RESPONSE\_UP | Details ... | 111 | 0.26 | 1.13 | 0.180 | 0.330 | 0.993 | 1391 | tags=23%, list=16%, signal=26% |
| 12 | HALLMARK\_REACTIVE\_OXYGEN\_SPECIES\_PATHWAY | Details ... | 37 | 0.29 | 1.00 | 0.441 | 0.584 | 1.000 | 1228 | tags=24%, list=14%, signal=28% |
| 13 | HALLMARK\_PI3K\_AKT\_MTOR\_SIGNALING | Details ... | 78 | 0.24 | 0.99 | 0.480 | 0.583 | 1.000 | 2040 | tags=37%, list=23%, signal=48% |
| 14 | HALLMARK\_KRAS\_SIGNALING\_DN | Details ... | 32 | 0.28 | 0.97 | 0.520 | 0.586 | 1.000 | 823 | tags=16%, list=9%, signal=17% |
| 15 | HALLMARK\_ADIPOGENESIS | Details ... | 152 | 0.22 | 0.97 | 0.580 | 0.559 | 1.000 | 918 | tags=15%, list=10%, signal=17% |
| 16 | HALLMARK\_PEROXISOME | Details ... | 65 | 0.23 | 0.89 | 0.704 | 0.706 | 1.000 | 1244 | tags=18%, list=14%, signal=21% |
Table: Gene sets enriched in phenotype **na **[plain text format]****
